# Long-term sky islands generate highly divergent lineages of a narrowly distributed stream salamander (*Pachyhynobius shangchengensis*) in mid-latitude mountains of East Asia

**DOI:** 10.1186/s12862-018-1333-8

**Published:** 2019-01-03

**Authors:** Tao Pan, Hui Wang, Pablo Orozcoterwengel, Chao-Chao Hu, Gui-You Wu, Li-Fu Qian, Zhong-Lou Sun, Wen-Bo Shi, Peng Yan, Xiao-Bing Wu, Bao-Wei Zhang

**Affiliations:** 1 Anhui Key Laboratory of Eco–engineering and Bio-technique, School of Life Sciences, Anhui University, Hefei, 230601 Anhui China; 2 School of Life Sciences, Anhui Normal University, Wuhu, 241000 Anhui China; 3 School of Biosciences, Cardiff University, Cardiff, UK; 4 Analytical and Testing Center, Nanjing Normal University, Nanjing, 210046 Jiangsu China

**Keywords:** *Pachyhynobius shangchengensis*, Sky island, Genetic diversity, Phylogeography, Population demography, Niche conservatism

## Abstract

**Background:**

Climate oscillation may have a profound effect on species distributions, gene flow patterns and population demography. In response to environmental change, those species restricted to montane habitats experienced expansions and contractions along elevation gradients, which can drive differentiation among sky islands.

**Results:**

The Shangcheng stout salamander (*Pachyhynobius shangchengensis*) is a cool stream amphibian restricted to high-elevation areas in the Dabie Mountains, East China. In the present study, we used mtDNA genes (Cyt *b* and *ND2*) of 193 individuals and 12 nuclear microsatellite loci genotyped on 370 individuals, representing 6 populations (JTX, KHJ, MW, TTZ, BYM and KJY) across the taxon’s distribution area, to investigate their genetic variation and evolutionary history of *P. shangchengensis*. Most populations showed unusually high levels of genetic diversity. Phylogenetic analyses revealed five monophyletic clades with divergence times ranging from 3.96 to 1.4 Mya. Accordingly, significant genetic differentiation was present between these populations. Bayesian skyline plot analyses provided that all populations underwent long-term population expansions since the last inter-glacial (0.13 Mya ~ 0.12 Mya). Msvar analyses found recent signals of population decline for two northern populations (JTX and KHJ) reflecting a strong bottleneck (approximately 15-fold decrease) during the mid-Holocene (about 6000 years ago). Ecological niche modelling has shown a discontinuity in suitable habitats for *P. shangchengensis* under different historical climatic conditions.

**Conclusions:**

Our results suggest that the niche conservatism of *P. shangchengensis* and sky island effects may have led to long-term isolation between populations. In sky island refuges, the mid-latitude Dabie Mountains have provided a long-term stable environment for *P. shangchengensis*, which has led to the accumulation of genetic diversity and has promoted genetic divergence.

**Electronic supplementary material:**

The online version of this article (10.1186/s12862-018-1333-8) contains supplementary material, which is available to authorized users.

## Background

Geological, climatic, and hydrological histories are the major drivers that have determined present-day patterns of biodiversity [1]. These factors can change the diversification at different temporal and geographical scales [2, 3]. For example, the uplifting of the Qinghai-Tibet Plateau caused habitat fragmentation, limitations to dispersal and the formation of gene flow barriers that accelerated divergence and even led to speciation [4, 5]. During the last million years, climatic oscillations, such as the glacial expansions and retreats, may have led to current patterns of species distributions and inter-population gene flow [6–8]. Hydrological histories might change dramatically under the influence of climatic oscillations and geological changes, which can influence the phylogeographic patterns drastically by the barrier effect, especially for aquatic organisms [9, 10]. In addition, during the Quaternary period, severe climatic oscillations made a profound impact on the phylogeography of species at mid-latitudes, especially for those endemic to mountainous areas [11, 12]. Therefore, the effects of geological, climatic, and hydrological histories were perhaps most pronounced in mid-latitude mountains where species adapted to local environmental conditions may have experienced alternating periods of isolation and connection during climatic fluctuations [10, 13–15]. The genetic consequences of these historical events are manifested in the phylogeographic patterns and levels of genetic variation across multiple taxa [6–8].

Sky islands are montane regions isolated from one another by intervening valleys with drastically different environmental conditions, in a similar way to how the sea separates oceanic islands [16]. In sky islands, changing climatic conditions can cause favorable environments for species or populations to expand or contract along an elevational gradient, leading to alternating periods of isolation and connection [6, 10–14, 17]. Species restricted to sky islands, especially aquatic taxa, often show unique patterns of population genetic structure [10, 13, 14, 17] influenced by historical climate-induced distributional shifts, and are highly vulnerable to future climate changes [6, 18]. Therefore, montane habitats often become hotspots of endemism, intraspecific genetic diversity and species richness, and provide the opportunity to study how different evolutionary processes lead to diversification [10, 12–14, 18, 19].

**Fig. 1:**
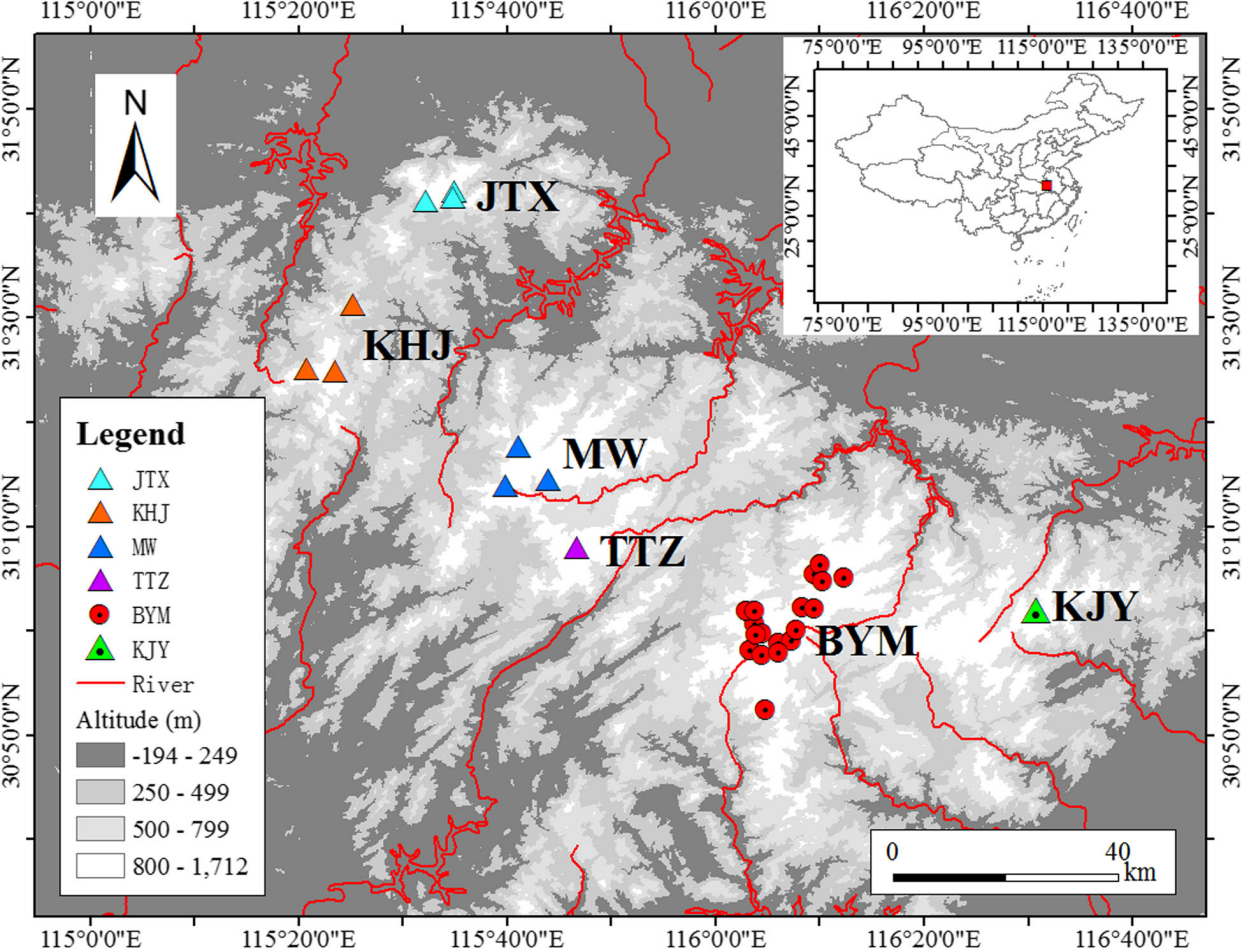
Sampling area and populations of *P. shangchengensis*. Sample sites are represented as triangles or dots in different colors. The map derived from https://glovis.usgs.gov/. The basic vector layers of rivers derived from http://www.resdc.cn/

The Dabie Mountains, located at mid-latitude in East Asia, are composed of a chain of ancient, isolated, low-middle elevation massifs (Fig. 1). The Dabie Mountains are located in the ecotone of subtropical evergreen broad-leaved forest and the warm-temperate deciduous broad-leaved forest zone. The climate in this region is transitional from subtropical to temperate, with annual average temperature of 12.5 °C and mean annual precipitation of 1832.8 mm [20]. The Shangcheng stout salamander (*Pachyhynobius shangchengensis*) is a stream salamander narrowly distributed in the Dabie Mountains and is endemic to the cool and oxygen-rich streams above 500 m [21]. Presently, *P. shangchengensis* is found distributed as six isolated areas/populations (Jiaoyuan-Tanghui-Xiaolongtan, JTX; Kangwangzhai- Huangbaishan- Jiufengjian, KHJ; Mazongling-Wochuan, MW; Tiantangzhai, TTZ; Baimajian-Yaoluoping-Mingtangshan, BYM; Kujingyuan, KJY) (Fig.1). In previous studies, distinct genetic divergence was found between some of these areas, however, these studies differed with respect to the mitochondrial gene and areas analysed (i.e. control region and JTX, KHJ, MW, TTZ, BYM [22], and Cyt *b*, COI and JTX, KHJ, TTZ, BYM [23]). In the mid-latitude areas, especially in mountains, species adapted to the local environment often show unique phylogeographic patterns, such as North American *Plethodon* [10, 13, 14]. Based on the above considerations, we have argued that a similar evolutionary course may also exist in the mid-latitude mountain areas of East Asia. In view of the distribution area characteristics, together with existing phylogeographic results for *P. shangchengensis*, we predicted that *P. shangchengensis* may be an ideal model organism to understand how climate change can impact genetic variation and phylogeographic patterns of species in mid-latitude mountains of East Asia.

Here we sampled *P. shangchengensis* throughout its range and used multiple molecular markers combined with GIS–based environmental niche analyses to better understand population genetic relationships and evaluate diversification processes. Firstly, we used statistical phylogenetic methods to test whether each geographically isolated sky island population is reciprocally monophyletic and whether the patterns of phylogenetic diversification identified are consistent with past climatic oscillation. Secondly, we examined whether the isolated sky island populations maintained genetic diversity effectively. Lastly, we used ecological niche modelling (ENM), combined with paleoclimate data to infer historical causes for the isolated distribution pattern of *P. shangchengensis.*

## Methods

### Sampling collection

Samples were collected from 370 individuals from 6 populations (JTX, KHJ, MW, TTZ, BYM and KJY), throughout the distribution range of *P. shangchengensis* between 2012 and 2015 (Fig. 1). Sampling involved capturing adults with dip nets and removing the tip of the tail (about 1 cm) prior to releasing the individuals. Samples were preserved in absolute ethanol in the field, and then stored at − 80 °C until used. For each population, we sampled no less than 20 individuals (Table 1). Individuals caught twice in different years (identified by their docked tails) were not sampled.

**Table 1:** Descriptive statistics for mitochondrial genes and 12 micorsatellite loci for *P. shangchengensis* populations in Dabie Mountains

Populations	Mitochondrial gene					Microsatellites				
	*N*	*Hd* ± SD	*π* ± SD	Tajima’s *D*	Fu’s *Fs*	*N*	*Na* ± SE	*H*_O_ ± SE	*H*_E_ ± SE	*F* ± SE
JTX	31	0.929 ± 0.030	0.0019 ± 0.0002	−1.326	−8.157*	53	8.83 ± 1.04	0.604 ± 0.074	0.702 ± 0.074	0.157 ± 0.064
KHJ	33	0.909 ± 0.032	0.0015 ± 0.0001	−1.221	−9.949*	48	9.25 ± 1.20	0.596 ± 0.088	0.699 ± 0.083	0.150 ± 0.063
MW	37	0.983 ± 0.010	0.0024 ± 0.0002	−1.645*	−20.902*	69	12.92 ± 1.13	0.687 ± 0.065	0.780 ± 0.068	0.107 ± 0.036
TTZ	35	0.983 ± 0.013	0.0029 ± 0.0003	−1.199	−19.588*	29	11.67 ± 1.14	0.724 ± 0.072	0.785 ± 0.066	0.147 ± 0.085
BYM	33	0.981 ± 0.015	0.0030 ± 0.0004	−1.781*	−16.555*	124	17.42 ± 1.10	0.756 ± 0.041	0.837 ± 0.041	0.095 ± 0.023
KJY	24	0.746 ± 0.071	0.0026 ± 0.0007	−0.627	−0.303	22	10.00 ± 1.12	0.696 ± 0.071	0.776 ± 0.051	0.120 ± 0.054
Total	193	0.990 ± 0.002	0.0345 ± 0.0004	−1.329	− 12.211	345	20.33 ± 1.44	0.693 ± 0.053	0.827 ± 0.059	0.077 ± 0.017

Note: *N* sample size, *Hd* haplotype diversity, π nucleotide diversity, *N*a number of alleles, *H*_O_ observed heterozygosity, *H*_E_ expected heterozygosity, *F* fixation index, *P* values of Tajima’s *D* test and Fu’s *Fs* test were calculated with 10,000 simulations, significant tests are indicated with an asterisk (* *P* < 0.01). The Tajima’s *D* test and Fu’s *F*s test of KJY population were conducted excluding the two KJY haplotypes clustered within the BYM population

### DNA extraction, sequencing, and microsatellite genotyping

Total DNA was extracted from samples using a standard proteinase K/phenol-chloroform protocol [22], and purified by EasyPure PCR Purification Kit (TransGene). Cytochrome *b* (Cyt *b*) and NADH dehydrogenase 2 (*ND2*) were amplified using primers designed from *Ranodon sibiricus* (NC004021) and *P. shangchengensis* (NC008080) mitogenome using Primer v5 [23] (Additional file 1: Table S1). PCR reaction mixtures (50 μL) consisted of 2 μL genomic DNA (concentration 10–50 ng/μL), 5 μL 10× buffer, 2 μL of 2.5 mM MgSO4, 4 μL of 2 mM dNTPs, 2 U *Taq* polymerase (TransGen Biotech, Beijing), 0.6 mM of each primer and sufficient pure molecular biology grade water. The amplification program included an initial denaturation step of 95 °C for 5 min followed by 32 cycles of denaturation at 95 °C for 30 s, primer annealing at 55 °C for 30 s and an extension at 72 °C for 80 s, with a final extension at 72 °C for 10 min. PCR products were purified using EasyPure PCR Purification Kit (TransGene), and sequenced on an ABI Prism 3730 automated sequencer using the BigDye Terminator v3.0 Ready Reaction Cycle Sequencing Kit (Applied Biosystems).

Twelve microsatellite loci were selected from previous studies (in Additional file 1: Table S1) [24]. PCR reaction mixtures (25 μL) consisted of 1 μL genomic DNA (concentration 10–50 ng/μL), 2 μL 10× buffer, 1 μL of 2.5 mM MgSO_4_, 2 μL of 2 mM dNTPs, 1 U *Taq* polymerase, 0.3 mM of each primer (forward primer fluorescently labeled with FAM, HEX or TAMRA) and sufficient water. The amplification program was conducted with following conditions: 5 min at 95 °C; followed by 35 cycles of 30 s at 95 °C, 20 s at the annealing temperature (Additional file 1: Table S1), 30 s at 72 °C; and 5 min at 72 °C. PCR products were visualized on an ABI 3730 semiautomated sequencer (PE Applied Biosystems) with the GS500 marker as size standard and then analyzed using GeneMarker 1.85 (version 1.3, SoftGenetics LLC) [25].

### Data analysis

Sequences were assembled using SeqMan in DNAStar [26]. All DNA sequences were aligned using Clustal X v.2.0 [27], trimmed, and deposited into GenBank (MF581931–MF582316). We estimated population nucleotide diversity (*π*) and haplotype diversity (*Hd*) in DnaSP v.5.10. Micro-Checker [28] was used to check the presence of null alleles and genotyping errors in microsatellite data. Deviation from Hardy-Weinberg equilibrium (HWE) for each locus and for all loci for each population was evaluated using exact probability tests implemented in GenePop v.4.2.1 [29]. Several population genetic summary statistics to describe genetic variation were estimated by GenAlEx v.6.5 [30] and GENETIX v.4.02 [31], including mean number of alleles per locus (*Na*), observed heterozygosities (*H*_O_), expected heterozygosities (*H*_E_), inbreeding coefficients (*F*) and pairwise *F*_ST_.

### Phylogeography and population structure

We reconstructed the phylogenetic relationships and divergence time of combined sequences (Cyt *b* and *ND2*) using BEAST v.1.8 [32], with *R. sibiricus* (NC004021) as outgroup. The best partition strategy and nucleotide substitution model for each data partition were determined with Partitionfinder v.2.1.1 [33]. The divergence times were estimated using a Bayesian Markov Chain Monte Carlo (MCMC) method with a strict clock method as implemented in BEAST. Due to the lack of suitable fossil calibrations for our analyses, we placed a lognormally distributed prior (centered at 40.2 Mya and with a 95% HPD between 34.5 and 46.2 Mya) on the age of the node marking the divergence between *P. shangchengensis* and the outgroup, based on the time calibrated molecular phylogenetic tree of Hynobiidae [34]. We simultaneously estimated topology and divergence dates by performing two independent runs of 20 million MCMC iterations sampling every 2000th iteration with the initial 25% of the samples discarded as burn-in. An uncorrelated lognormal model of lineage variation with an expansion growth tree prior and a strict molecular clock was used [32] based on the model comparisons carried out in Tracer v.1.6 [35] including a comparison with the alternative relaxed molecular clock model. Convergence of the two independent MCMC runs was assessed in Tracer, as were convergence of model parameter values (effective sample size [ESS]) to ensure ESS values > 200. The tree and posterior distribution were summarized with TreeAnnotator v.1.8 [36] and visualized in FigTree v.1.4.3 [37].

A minimum-spanning network based on mitochondrial genes was created with Network v.4.6.1 (Fluxus Technology, Suffolk, UK), using the full median-joining algorithm [38] to visualize relationships of the mitochondrial DNA (mtDNA) haplotypes among localities. Genetic differentiation (*F*_ST_) between populations was estimated in Arlequin v.3.5.1.3 [39] based on haplotype frequency differences and with 10,000 permutations to assess significance. Homogeneity of haplotype distributions among localities was tested using a single-level AMOVA in Arlequin.

A Bayesian analysis of population structure using STRUCTURE [40] was carried out to estimate the number of potential clusters present in the microsatellite data and to assign individuals to inferred clusters. Ten independent runs were carried for different values of K (the number of clusters) between 1 and 8, using no prior information about individual location, and assuming admixture and correlated allele frequencies. The MCMC analyses were run for a total of 1 million generations discarding the first 100,000 as burn-in. The most likely K explaining the variation in the data was selected by choosing the K result for which the log likelihood [Ln Pr(X/K)] of the posterior probability of the data was the highest [40], and the ΔK statistic [41]. The results were graphically displayed with the software DISTRUCT [42].

A spatial cluster analysis was conducted with the Geneland package [43] in R 3.3.1 (R Development Core Team 2016). The MCMC iterations were set to 100,000 steps sampling every 100th iteration and discarding the first 10% iterations as burn-in period. K values between 1 to 6 were tested with individuals assigned to one K cluster (1 ≤ K ≤ 6) based on their multilocus genotypes and spatial coordinates. To confirm that Geneland was long enough, we carried out 10 different runs and compared the parameter estimates between them (K, individual population membership, maps). The best result was chosen based on the highest average log posterior density.

### Population demography

Mismatch distributions were estimated to test the null hypothesis of recent population growth using Arlequin. A goodness-of-fit test was used to determine the smoothness of the observed mismatch distribution (using Harpending’s raggedness index, Rag) and the degree of fit between the observed and simulated data (using the sum of squares deviation, SSD) [44]. Due to their sensitivity to demographic changes, we also estimated Tajima’s *D* [45] and Fu’s *Fs* [46] using 10,000 coalescent simulations to assess significance in Arlequin. We also examined past population dynamics of all phylogeographic lineages of *P. shangchengensis* using the Bayesian skyline plots (BSP) with the model selected by Partitionfinder. BSP were performed in BEAST to reconstruct historical population size dynamics by using the same parameter sets as previous divergence time estimation. Each dataset was run four times for 20 million MCMC iterations, sampling every 2000 iterations and discarding the first 25% of the iterations as burn-in. Tracer was used to check the convergence of the MCMC analyses (ESS values > 200).

The software Msvar v.1.3 [47] was used to characterize the recent demographic history of the six populations of *P. shangchengensis* using the microsatellite data. The method assumes that a current population (of size *N*_0_) passed through a demographic change (a bottleneck or an expansion) at time *T* in the past, which changed its size from *N*_1_ to *N*_0_ following an exponential model. The coalescent simulations were conditioned with various prior distributions to assess the lack of dependency of the posterior estimates of the three inferred parameters (*N*_0_, *N*_1_ and *T*). The prior and hyperpriors used in Msvar were showed in Additional file 2: Table S2. The prior distributions tested consisted of (i) a model in which both *N*_0_ and *N*_1_ had the same prior upper bound, (ii) where *N*_0_ had a wider prior than *N*_1_ (i.e. an expansion was allowed) and (iii) where *N*_1_ had a wider prior than *N*_0_ (i.e. a bottleneck was allowed). In addition, various upper bound values for the prior of the time of change were also tested. As to the microsatellite mutation rate, we used an average vertebrate microsatellite mutation rate of 10^− 4^ [48] allowed to vary across our markers between approximately 10^− 2^ and 10^− 6^ (Additional file 2: Table S2). The generation time of 6 years was selected based on previous results [49]. Each Msvar run consisted of 2 × 10^9^ iterations of the MCMC algorithm discarding the first 10% of the coalescent simulations as burn-in. Convergence of the chains was assessed with Gelman and Rubin’s diagnostic [50] using the CODA library [51] implemented in R based on the four runs performed for each populations with different prior distributions.

### Niche reconstruction

We use ENM to simulate the distribution pattern of *P. shangchengensis* under different climate backgrounds using climatic data and GPS coordinates of 30 distribution localities using the maximum entropy algorithm in the program MAXENT v. 3.3.3 k [52]. For the current climate predictions, we downloaded raster coverages of 19 environmental climatic variables from the WorldClim database (http://www.worldclim.org) at 30 arc-seconds resolution (~ 1 km^2^) and clipped these coverages to a region that encompassed the entire Dabie Mountains’s range (30 ~ 32°N, 114 ~ 117°E) [53]. We performed a correlation analysis between the nineteen climatic variables with ArcGIS 10.0 (ESRI) in the defined area, and then selected a subset of eight variables with a correlation ® lower than 0.85: BIO3 (Isothermality), BIO5 (Max Temperature of Warmest Month), BIO8 (Mean Temperature of Wettest Quarter), BIO9 (Mean Temperature of Driest Quarter), BIO13 (Precipitation of Wettest Month), BIO14 (Precipitation of Driest Month), BIO15 (Precipitation Seasonality), BIO18 (Precipitation of Warmest Quarter). Assuming niche conservatism over time [54], we projected the species distribution model to the climatic data layers for current environmental conditions, and for layers describing climatic variables during the mid-Holocene (6000 years ago), the last glacial maximum (LGM, about 22,000 years ago) and the last inter-glacial (LIG, 0.13 Mya ~ 0.12 Mya) [55], to obtain the predicted species distribution across time. For the LIG, LGM and the mid-Holocene predictions we used the same set of bioclimatic variables at a maximum resolution (2.5 arc minutes) was collected from the WorldClim database. The community climate system model (CCSM) [56] were used to reconstruct the paleodistribution.

We performed 15 replicates for the model with subsamples, 10,000 background points and 25% of points set for random testing. Analyses were run using default program parameter cumulative outputs, a 0.00001 convergence threshold, and a maximum of 5000 iterations. The area under the curve (AUC) of the receiver operating characteristic (ROC) plot were used for model evaluation [52]. All models were post processed and visualized in ArcGIS 10.0 (Esri, Redlands, CA, USA).

## Results

### Genetic diversity

We sequenced 984 bp of the *ND2* gene and 1127 bp of the Cyt *b* gene from 193 individuals. Total *Hd* and *π* across all populations were 0.990 and 0.0345, respectively (Table 1). The TTZ and MW populations exhibited the highest haplotype diversity (*Hd* = 0.983) while the KJY population possessed the lowest value (*Hd* = 0.746). The BYM population exhibited the highest nucleotide diversity (*π* = 0.0030) while KHJ presented the lowest one (*π* = 0.0015).

For microsatellite data, 345 individuals were successfully genotyped at twelve loci (Table 1). No consistent departure from HWE or linkage disequilibrium was observed across the six populations. The nuclear genetic diversity varied between populations and summary statistic used with *N*a ranging from 8.83 (JTX) to 17.42 (BYM), *H*_O_ ranging from 0.596 (KHJ) to 0.756 (BYM), while *H*_E_ varied from 0.699 (KHJ) to 0.837 (BYM). Overall, among the six populations, BYM showed higher genetic diversity than the other five populations (Table 1).

### Phylogenetic reconstructions

**Fig. 2:**
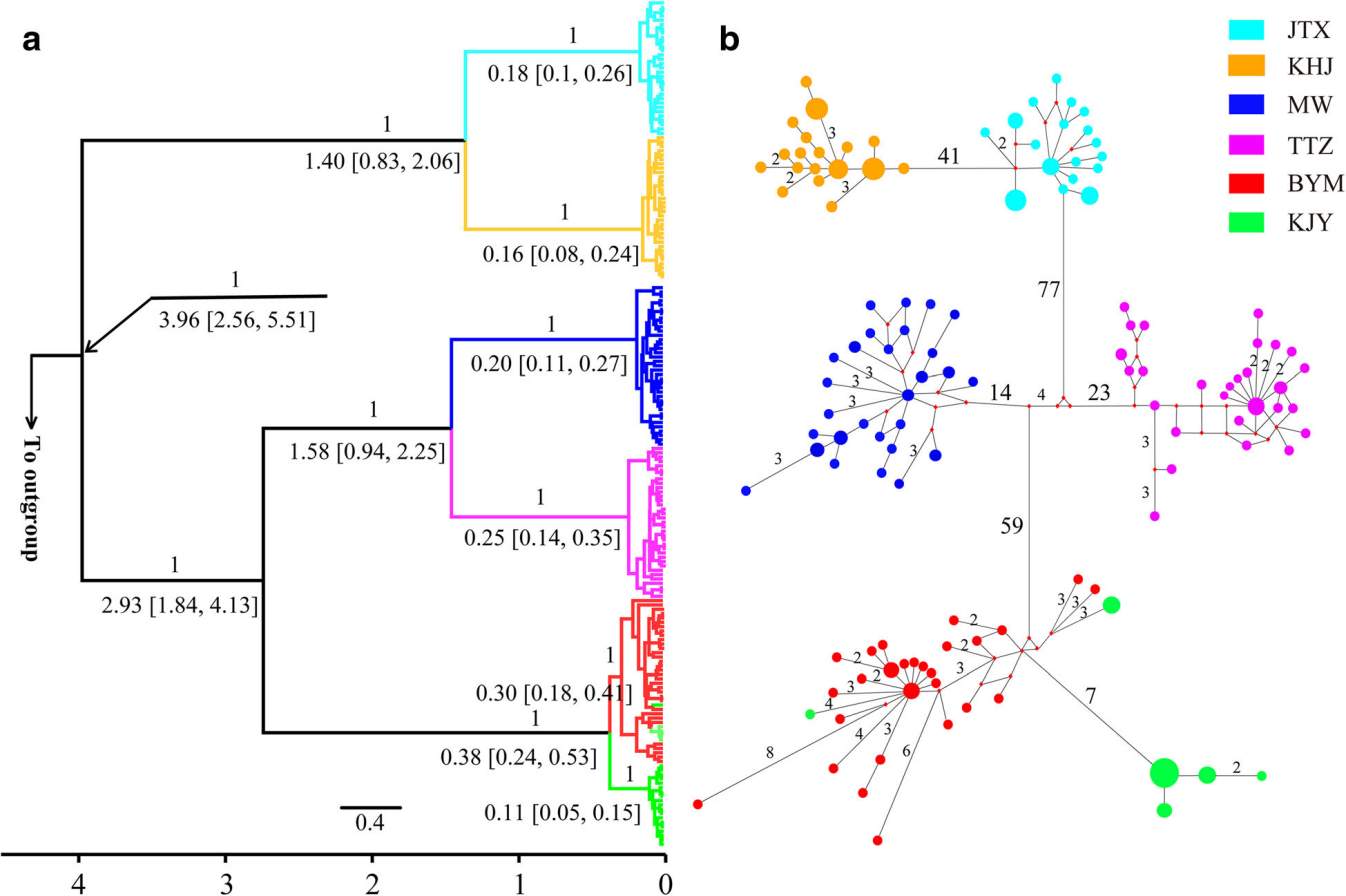
Phylogenetic relationships and haplotype network based on the mtDNA haplotypes of *P. shangchengensis*. **a** Phylogenetic tree of *P. shangchengensis* based on mitochondrial data. The numbers above branches are Bayesian Posterior Probabilities (BPP) indicating node support and the numbers underneath branches are split time estimates (Mya) with their 95% highest posterior density in square brackets; **b** Median-joining network with node sizes proportional by area to the frequencies of haplotypes. The numbers of mutations separating the haplotypes are shown on the branches, except for the one-step mutations. The little red diamond nodes indicate undetected haplotypes

Partitionfinder identified that the most suitable models of evolution for the two mitochondrial genes studied here were HKY + I model for the combination of the first and second codon positions of both genes, while the GTR + G model for the third codon positions. The phylogenetic tree was split into two major branches, which corresponded to the northern two populations (JTX, KHJ) and southern four populations (MW, TTZ, BYM, KJY) respectively (Fig. 2). In the northern branch, the individuals from JTX and KHJ populations formed two distinct clades respectively, which acted as sister groups for each other. The southern branch was composed of three clades, with the individuals of MW and TTZ populations forming distinct monophyletic clusters respectively. The third southern clade was formed by the individuals of the BYM and KJY populations, however, these could not be completely differentiated form each other as the clade with all the BYM individuals also included some KJY individuals (Fig. 2). The network analysis recapitulated the phylogenetic tree and had five haplogroups with no shared haplotypes among different populations except for BYM and KJY. These five haplogroups are separated by multiple mutations (Fig. 2). The estimated time to the most recent common ancestor (TMRCA) of the entire ingroup was 3.96 Mya (95% HPD, 2.56–5.51 Mya), with the five lineages originating between 2.93 and 1.4 Mya (Fig. 2).

### Population structure

**Fig. 3:**
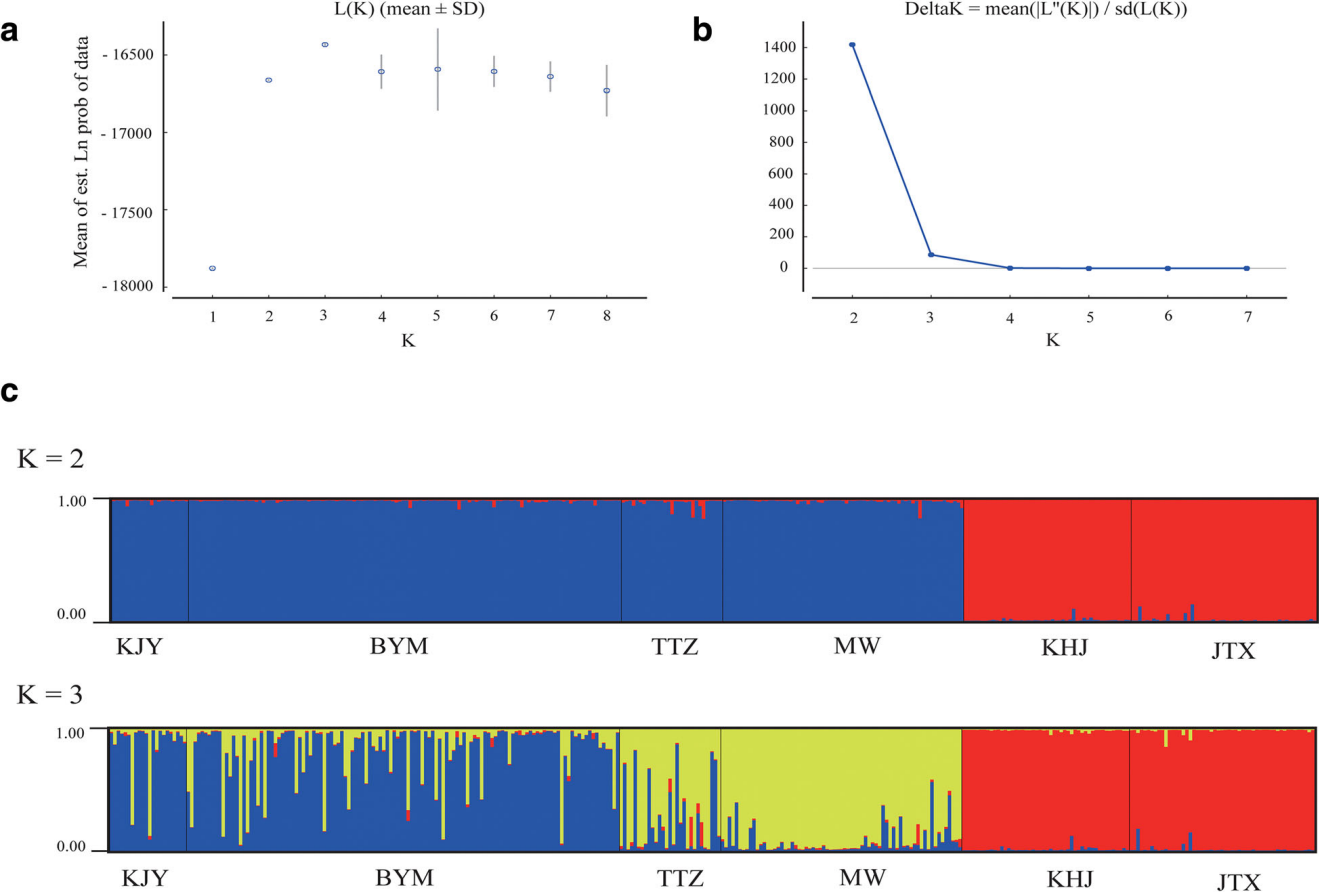
STRUCTURE clustering results based on microsatellite genotype data of six *P. shangchengensis* populations. **a** The linear relationship between LnP(D) and the number of clusters (K); **b** ΔK values as a function of K based on 5 runs; **c** STRUCTURE output for K = 2 and 3

**Fig. 4:**
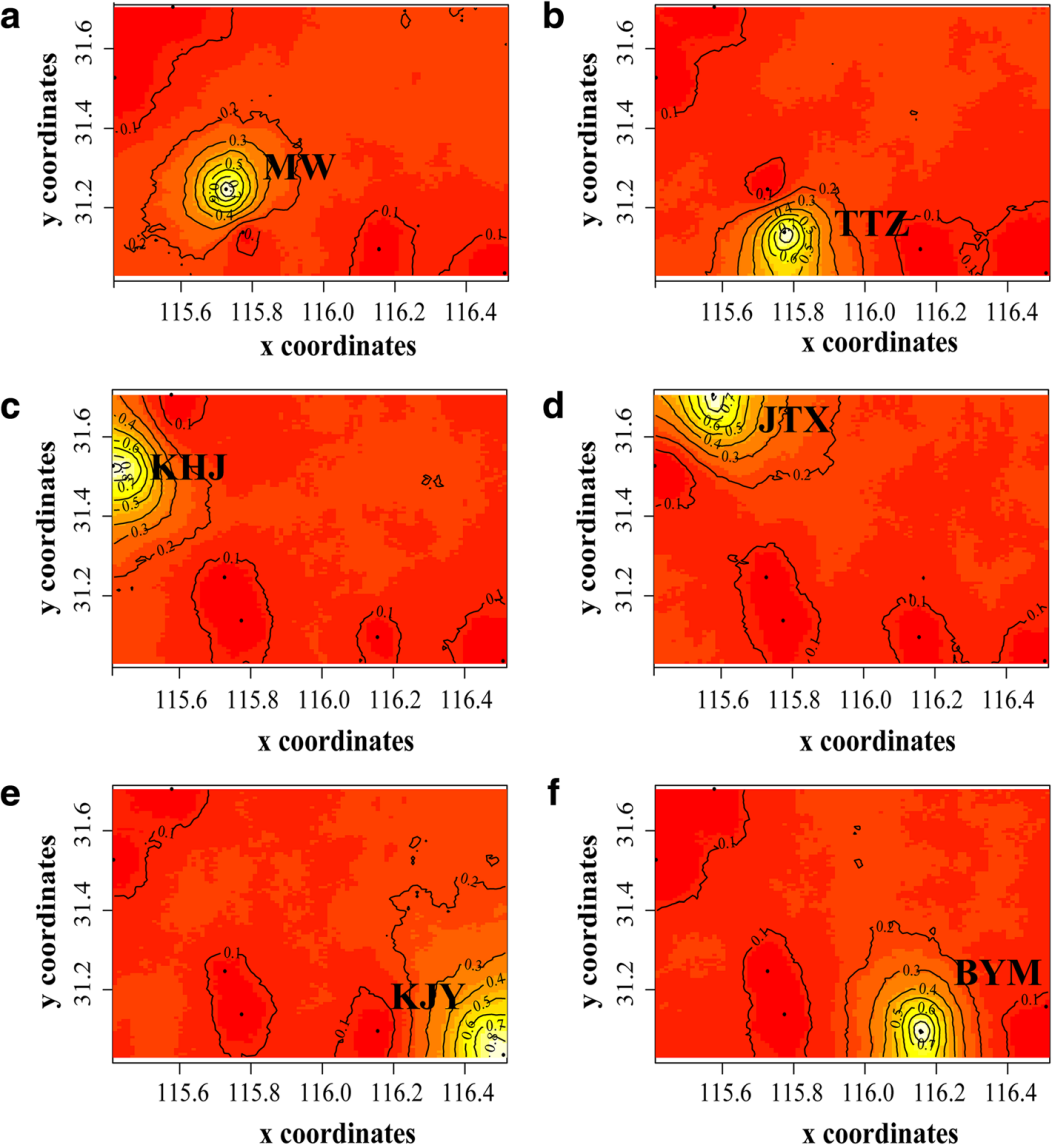
Spatial distribution of each group defined by Geneland for K = 6. **a** Cluster 1, **b** Cluster 2, **c** Cluster 3, **d** Cluster 4, **e** Cluster 5, and **f** Cluster 6. Clusters are indicated by areas with different intensities of color. Lighter-colored areas indicate a higher probability that individuals belong to that cluster

The *F*_ST_ values among populations were all significant and ranged from 0.494 (BYM and KJY) to 0.962 (JTX and MW) for mtDNA data and from 0.087 (MW and TTZ) to 0.590 (JTX and KJY) for the microsatellite data (Table 2), indicating moderate to high level of genetic differentiation among populations. STRUCTURE analyses revealed a maximum ΔK value for K = 2 supporting the split into two main branches observed with the mitochondrial DNA (Fig. 3). However, it is worth noting that the STRUCTURE results showed a maximum posterior probability for K = 3 (Fig. 3) supporting the split of the southern branch observed with the mitochondrial data into a cluster formed by the MW and TTZ individuals, and another one formed by the BYM and KJY individuals. These two southern clusters share a substantial amount of the genetic variation as reflected by the admixture between individuals in both clusters (Fig. 3). Further splitting of the samples with STRUCTURE for K values of 4 to 6 does not result in better clustering results (Additional file 3: Figure S1). The inclusion of the geographical prior into the spatial model of Geneland with the microsatellite data consistently identified six groups in the ten replicas of the analysis carried out and which correspond to the six populations in this study (Fig. 4 and Additional file 4: Table S4).

**Table 2:** Pairwise *F*_ST_ among six populations of *P. shangchengensis* based on mtDNA sequence (below diagonal) and microsatellite data (above diagonal), respectively

	KJY	BYM	TTZ	MW	KHJ	JTX
KJY		0.119*	0.293*	0.252*	0.520*	0.590*
BYM	0.494*		0.151*	0.143*	0.432*	0.474*
TTZ	0.927*	0. 925*		0.087*	0.359*	0.418*
MW	0.934*	0. 931*	0.891*		0.426*	0.475*
KHJ	0.954*	0. 949*	0.951*	0.954*		0.218*
JTX	0. 961*	0.956*	0.958*	0.962*	0.923*	

Note: Significant tests are indicated with an asterisk (*P < 0.01)

The AMOVA analysis of the mitochondrial data with the predefined grouping of the samples according to the three clusters suggested by STRUCTURE (i.e. JTX and KHJ, MW and TTZ, BYM and KJY) shows that 61.02% of the genetic variation occurs among groups, 33.83% among populations within groups and 5.15% within populations (Additional file 5: Table S3). The same analysis performed on the microsatellite data shows that 7.75% of the genetic variation occurs among groups, 2.8% among populations within groups, and 89.46% within populations (Additional file 5: Table S3).

### Historical demography

The neutrality test (Tajima’s *D* and Fu’s *Fs*), mismatch distribution analysis and BSPs were conducted on the mtDNA sequence data and excluding the two KJY haplotypes clustered within the BYM population. Fu’s *Fs* and Tajima’s *D* were negative in all six populations, however, while *Fs* was significant for all populations except KJY, only MW and BYM presented a significant *D* (Table 1). This results are further supported by the unimodal mismatch distributions observed in four out of six populations (TTZ and KJY showed multimodal or nearly unimodal distributions; Additional file 6: Figure S2 and Additional file 7: Table S5), and by the BSP results that showed that all populations experienced a demographic expansion (Additional file 8: Figure S3).

**Fig. 5:**
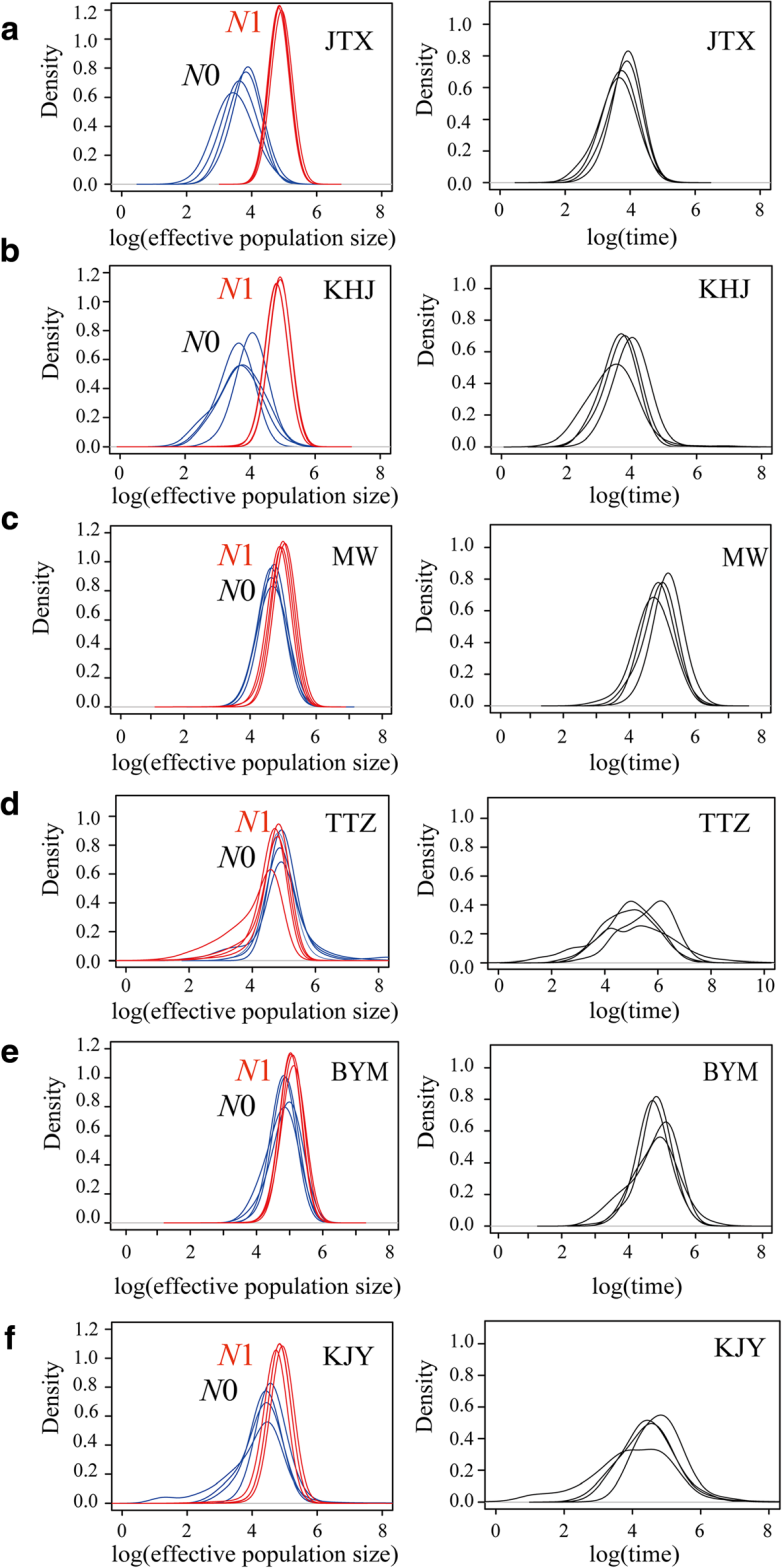
Posterior distributions of the demographic parameters inferred with Msvar of six populations based on microsatellite data (**a**–**f**). Estimated posterior distributions of current (*N*_0_, blue curve) and ancestral (*N*_1_, red curve) effective population sizes and time since population change (T, black curve) on a logarithmic scale based on a generation time of 6 years

The Msvar analysis based on microsatellite genotype data was run four times independently under various demographic models. The Gelman and Rubin statistic inferred from these runs were smaller than the upper threshold of 1.2 for all populations indicating convergence of the MCMC. The Msvar results for the southern populations supports a stable demography in these populations with *N*_0_ and *N*_1_ posterior distributions with their modes almost completely overlapping each other. Contrastingly, the northern populations (JTX and KHJ) have an *N*_0_ (mode ~ 4870 and 5959, respectively) almost 15 times smaller than their *N*_1_ (mode ~ 74,989 and 81,752, respectively) indicating that these populations passed through a drastic bottleneck approximately 6000 years ago (Fig. 5, Table 3).

**Table 3:** Parameters Msvar analysis of each population in *P. shangchengensis*. Posterior probabilities and 95% highest posterior intervals for the parameters inferred in Msvar

Population	log_10_(*N*_0_) ± SD	*N*_0_	log_10_(*N*_1_) ± SD	*N*_1_	*N*_1_/*N*_0_	log_10_(T) ± SD	T (year)
JTX	3.69 ± 0.22	4870	4.88 ± 0.05	74,989	15.40	3.80 ± 0.12	6310
KHJ	3.78 ± 0.15	5957	4.91 ± 0.02	81,752	13.72	3.80 ± 0.32	6310
MW	4.70 ± 0.04	50,119	4.96 ± 0.06	91,725	1.83	4.95 ± 0.19	89,125
TTZ	4.86 ± 0.08	72,862	4.73 ± 0.03	53,088	0.73	5.38 ± 0.63	237,137
BYM	4.88 ± 0.03	74,989	5.09 ± 0.05	122,321	1.63	4.90 ± 0.16	79,433
KJY	4.44 ± 0.13	27,384	4.81 ± 0.10	64,938	2.37	4.61 ± 0.27	40,973

*N*_0_ current effective population size, *N*_1_ historical effective population size, T time of the bottleneck

### Ecological niche modeling

**Fig. 6:**
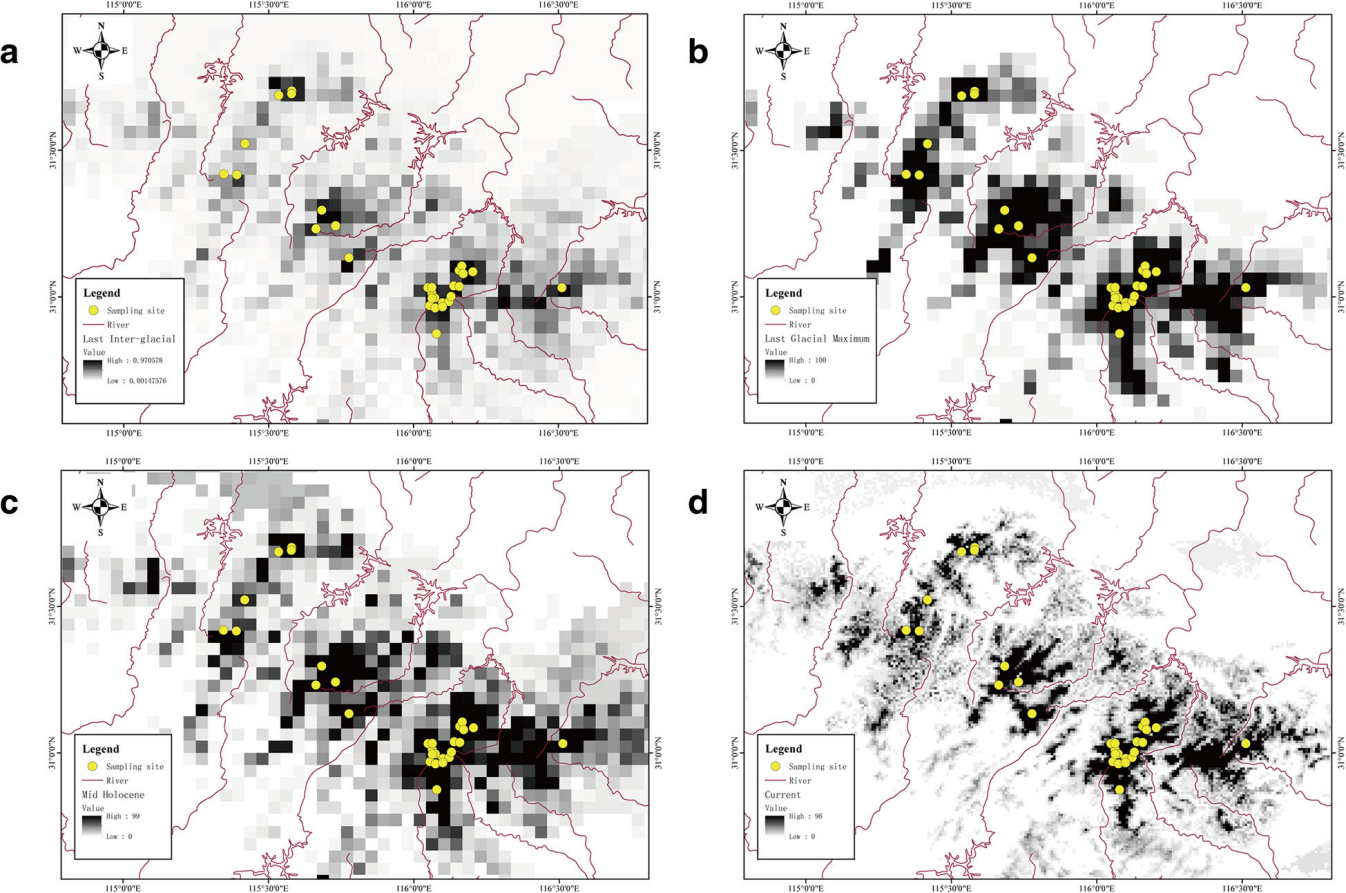
Ecological niche modeling of *P. shangchengensis* under (**a**) Last Inter-glacial conditions, (**b**) Last Glacial Maximum conditions, (**c**) Mid Holocene, (**d**) Current conditions. Location data used for modeling are indicated as yellow dots

The output of MaxEnt consists of a grid map with each cell having an index of suitability between 0 and 1. The predicted distribution of *P. shangchengensis* based on ecological niche modeling closely matched its actual distribution (Fig. 6d), differing significantly from random (AUC = 0.998; 25% of data points set for random testing). Most sampling localities in this study fell within the area of highest predicted suitability. Of the 8 environmental variables used to construct the niche model, BIO5 (Max Temperature of Warmest Month), BIO9 (Mean Temperature of Driest Quarter) and BIO8 (Mean Temperature of Wettest Quarter) had the highest contributions to the model (69.1, 18.3 and 5.5%, respectively) (Additional file 9: Table S6). During LIG (0.13 Mya ~ 0.12 Mya), the potential distribution was the smaller than that of other three periods (Fig. 6a), and the distribution during mid Holocene (about 6000 years before the present) (Fig. 6c) of *P. shangchengensis* was smaller than that during the LGM (about 22,000 years before) (Fig. 6b). Based on the predictions of ENMs, although the distribution area has changed under different historical periods and climatic conditions, the results showed long-term stability in the availability of suitable habitat and long-term isolation among population distribution area by unsuitable environment at low altitude in *P. shangchengensis* (Fig. 6a, b, c, d)*.*

## Discussion

### Genetic diversity

Small and isolated populations for prolonged periods often encounter a number of genetic risks, such as the loss of genetic variation due to genetic drift, inbreeding the potential accumulation of weakly deleterious mutations [57–59]. In this study, we described *P. shangchengensis* populations that have been isolated from each other for more than one million years, however, each populations still harbored relatively high levels of genetic diversity when compared to other narrowly distributed salamanders, such as *Batrachuperus tibetanus* (Hynobidae, Caudata) [60] and various Plethodontids (Plethodontidae, Caudata) such as *P. ouachitae*, *P. fourchensis* and *P. caddoensis* [10, 13, 14], or even some widely distributed amphibians such as Hensel’s swamp frog (*Pseudopaludicola falcipes*) in South America’s Pampas or Southwest China’s Leishan spiny toad (*Leptobrachium leishanense*) [61–63]. Several theories have been put forward to explain the high genetic diversity pattern observed in some species, such as species’ historic levels of genetic diversity, larger extant population size, longer generation times, random mating, gene flow, no inbreeding and lack of bottlenecks [64]. In general, during the Quaternary, dramatic climate oscillation led to expansions and contractions of glaciated areas, having an important impact on the montane habitats and leading some of them to become hotspots of genetic diversity [11, 12]. Based on the ENM prediction (Fig. 6), the Dabie Mountains could have provided a stable habitat for *P. shangchengensis* under different climate condition during the LIG, LGM and mid-Holocene periods, probably through the presence of favorable environments in long-term and stable montane stream habitats that prevented the collapse of most of its populations and thus favored the maintenance of their genetic variation.

In this study, a relatively low genetic diversity was observed in the JTX and KHJ populations. The amount of genetic variation may be correlated with the suitable habitat size for a population [4, 12, 17, 18, 65–67], with JTX and KHJ showing smaller current and past suitable habitat sizes in comparison to the other southern populations of *P. shangchengensis*. However, this observation is probably exacerbated by the demographic history of these two populations that passed through a relatively recent and strong bottleneck during the mid-Holocene further leading to a decrease in their genetic variation in comparison to the southern populations (Fig. 5, Table 3).

### Geographic structure

Species restricted to montane sky island habitats often show high levels of inter-population genetic divergence and unique patterns of genetic structure [14, 19, 68–70], a pattern magnified in species distributed in mid to high-latitude mountain regions [11, 12]. In such habitats at high elevation, populations are often separated from each other by unsuitable habitat at lower elevation areas due to niche conservatism [10, 13, 14, 16, 17, 71]. This separation was often facilitated by climatic contrasts at different altitudes or orogenesis with the lower elevation areas becoming geographic barriers to gene flow leading to habitat fragmentation [16]. Consequently, species that moved along the altitudinal gradient, probably as a consequence of climate changes, may have originally formed a continuous population at lower altitudes that becomes subdivided when entering the sky islands and over time results in monophyletic groups through genetic drift [16, 17]. For example, in western Arkansas (USA), the unique physiographic features of the Ouachita Mountains area, coupled with species response to climatic factors, drove lineage divergence in three *Plethodon* species (*P. ouachitae*, *P. fourchensis* and *P. caddoensis*) resulting in the classic phylogeographic structure associated with stream drainages and mountains [10, 13, 14]. *B. tibetanus* (Hynobiidae, Caudata) is a typical stream salamander in the Qinling Mountains showing a split into two deeply divergent lineages that formed in the past 3 to 4 Mya as a response to the orogenesis of Qinling Mountains during the late Cenozoic [60].

In the Quaternary, sharp climatic oscillations caused species at mid-latitudes to experience the most severe population expansions and contractions [6]. In the mid-latitude mountain areas, the changes were also reflected in altitude [11, 12]. However, in East China, the coastal mountains intercept moisture and heat, transported by monsoons from the ocean and provide relative climatic and ecological stability [72–75]. The Dabie Mountains, composed of a chain of ancient isolated low-middle elevation massifs (Fig. 1) and located in a mid-latitude area of East Asia, are believed to have been able to maintain a relatively stable climate over the last several million years [73, 76]. Based on the ENMs prediction results, although the distribution area of *P. shangchengensis* has changed over different historical periods and climatic conditions, there has always been extensive suitable habitat for different populations (Fig. 6), even during the LGM that marked the most drastic climate oscillations of the whole Pleistocene [77]. The ENM results show that in lower elevation areas, unsuitable habitat for *P. shangchengensis* always existed, acting as a strict and effective isolation barrier for this species with strict niche conservatism. Once these discontinuous sky islands were formed, deep inter-population genetic divergences of *P. shangchengensis* accumulated gradually. Consequently, it is likely that an ancient *P. shangchengensis* population became fragmented after entering the current species distribution range with unsuitable habitat characterizing the geographic space between sky islands and having played a major role in the divergence of these populations.

### Population demography

Generally, mid-latitude regions, and especially mountainous areas, experienced the most significant climatic changes due to the fluvial and glacial-dominated conditions during the Quaternary [11, 12]. In the mid-latitude regions of Europe and North America, the distributions of organisms were affected by drastic climate oscillations and glacial cycles during this period [6, 78]. Compared with the climate in Europe and North America, the mid-latitude regions in East Asia area were a mosaic of mountains lower than 2000 m characterized by a relatively mild Pleistocene climate due to a lack of ice sheets in many areas [73, 75, 79]. Although previous studies have suggested the glaciations during the Quaternary in East Asia were responsible for the cyclic population expansions or contractions for several taxa [80–82], evidence is available that for others (e.g. spiny frogs – *Quasipaa boulengeri* [83]) these events had a little or no limited, if any, effect. In this study, based on *P. shangchengensis* mtDNA data a long-term demographic expansion since the LIG was observed (0.13 Mya ~ 0.12 Mya) (Additional file 8: Figure S3) [84]. This observation agrees with the ENM prediction models for the species that show a relatively smaller distribution area at the beginning of LIG and relatively wide range of suitable habitats during LGM (Fig. 6). The onset of the demographic expansion during the LIG and population stabilization during the last glaciation of *P. shangchengensis* are in accordance with the observations for many other species in East Asia, such as the tufted deer (*Elaphodus cephalophus*) [82], the Chinese Hwamei (*Leucodioptron canorum*) [85] and the Chinese bamboo partridge (*B. thoracica*) [81]. In mid-latitude Europe, various reptiles also experienced similar demographic changes, e.g. the Italian and Maltese wall lizards (*Podarcis siculus* and *P. filfolensis*, respectively) [77, 86]. Obviously, some temperate species in mid-latitude regions, especially those insensitive to environmental changes, underwent attenuated glacial population contractions, or may have passed through population expansion [77, 81, 82, 85, 86].

In the present study, all *P. shangchengensis* populations, except the two northern populations (JTX and KHJ), remained stable during the Holocene (Fig. 5). The two northern populations showed evidence of a strong population decline approximately 6000 years ago (Fig. 5, Table 3). The decline of those two populations may be attributed to drastic climatic changes in the mid-Holocene that affected the northern boundary of the Dabie mountains more strongly that the rest of this southern range. Several recent studies highlight the mid-Holocene as a period of particularly profound climate variation, expressed as the decline of land air temperatures across much of the globe [87–89]. Additionally, compared with the habitat of four southern populations (MW, TTZ, BYM, KJY), the KHJ and JTX populations occupy a relatively smaller effective habitat area (Fig. 1) making them more sensitive to environmental changes in their geographic range as smaller populations have higher risk of population decline or extinction [90, 91].

## Conclusion

Mid-latitude mountains are unique environments where species may exhibit amazing genetic divergence and population demography even in narrow areas. In this study, we show how the genetic variation of *P. shangchengensis* has been shaped by a combination of factors including the region’s topography, historical climate conditions and micro-climatic zones. The niche conservatism of *P. shangchengensis* within the context of its distribution range and evolutionary history support the sky island effect in the Eastern China Dabie Mountains. Although the current genetic variation in the species does not show signs of a population decline in modern times, the fragility and susceptibility to modern climate change of the environment it inhabits argues in favor of developing a management strategy for this and other species experiencing the sky island effect in the region.

## Additional files

Additional file 1: **Table S1.** Details on the primers for amplified the *ND2* gene, Cyt *b* gene and twelve microsatellite loci of *P. shangchengensis*. (DOCX 19 kb)

Additional file 2: **Table S2.** Prior and hyperpriors used in Msvar based on microsatellite data. (DOCX 17 kb)

Additional file 3: **Figure S1.** STRUCTURE clustering results for K = 4, 5 and 6. (TIF 2521 kb)

Additional file 4: **Table S4.** Multiple runs for inferring the number of populations of *P. shangchengensis* with Geneland. Bold indicates the highest average posterior probability. (DOCX 16 kb)

Additional file 5: **Table S3.** Results of hierarchical AMOVA based on mtDNA and microsatellites. Groups are set as JTX and KHJ, MW and TTZ, BYM and KJY. (DOCX 17 kb)

Additional file 6: **Figure S2.** Mismatch distributions analyses for the six populations of *P. shangchengensis* based on the mtDNA sequence data (a–f). The two KJY individuals clustering with BYM were excluded from the demographic history analyses. The x coordinate represents the number of differences between pairs of sequences and the y coordinate represents the frequencies of pairwise differences. The blue histograms are the observed frequencies of pairwise divergences among sequences and the green line refers to the expected shape of the distribution under the model of population expansion. (TIF 2468 kb)

Additional file 7: **Table S5.** Results of demographic statistics of *P. shangchengensis* based on mtDNA data. (DOCX 18 kb)

Additional file 8: **Figure S3.** Bayesian skyline plot of effective population size in *P. shangchengensis* based on the mtDNA data (a–f). The BSP analyses of the KJY population was calculated excluding the individuals with two haplotypes clustered more closely to the BYM population. The x-axis indicates time in Mya BP, and the y-axis indicates the effective population size in units of *N*_eτ_ (the product of effective population size and generation time in Mya). The blue areas represent 95% highest posterior density. Time is expressed in million years. (TIF 2011 kb)

Additional file 9: **Table S6.** Results from Principal Components Analysis on environmental variables used in comparison of environments among occurrence localities for *P. shangchengensis* and the relative contribution of each variable to the niche model. (DOCX 16 kb)

## Abbreviations

BSP: Bayesian skyline plots
BYM: Baimajian-Yaoluoping-Mingtangshan
CSM: Community Climate System Model
Cyt *b*: Cytochrome *b*
ENM: Ecological niche modelling
JTX: Jiaoyuan-Tanghui-Xiaolongtan
KHJ: Kangwangzhai-Huangbaishan- Jiufengjian
KJY: Kujingyuan
LGM: Last Glacial Maximum
LIG: Last Inter-glacial
MCMC: Markov Chain Monte Carlo
MW: Mazongling-Wochuan
*ND2*: NADH dehydrogenase 2
TMRCA: Time to most recent common ancestor
TTZ: Tiantangzhai
