# Supplementary material for: Long-term sky islands generate highly divergent lineages of a narrowly distributed stream salamander (Pachyhynobius shangchengensis) in mid-latitude mountains of East Asia
Source: BMC Evol Biol. 2019 Jan 3;19:1. doi: 10.1186/s12862-018-1333-8 (PMC6318985; doi:10.1186/s12862-018-1333-8)
Supplement: Supplementary file 1 — Table S1. Details on the primers for amplified the ND2 gene, Cyt b gene and twelve microsatellite loci of P. shangchengensis. (DOCX 19 kb) [file 12862_2018_1333_MOESM1_ESM.docx]

**Table S1** Details on the primers for amplified the *ND2* gene, Cyt *b* gene and twelve microsatellite loci of *P. shangchengensis*.

| Primer type | Pair | Name | Sequence (5’–3’) | Annealing temperature |
| --- | --- | --- | --- | --- |
| *ND2* Primer | 1 | PS-ND2-F1230 | CTTCCACTAATGAGCCCTTATGCA | 53 |
|  |  | Sp-ND2-R | TTACAAGGGCTAGAAGATTTTAACTTC | 53 |
| Cyt *b* Primer | 2 | PS-Cytb-F | TAACCAGGACCTTTGACTTGAA | 53 |
|  |  | PS-Cytb-R | AGATGAATGTACTATATACATAATATGAC | 53 |
| Microsattlite Primer | 1 | Psh29-F | TAMRA - ACAGATTGCTGCAACAGAG | 60 |
|  |  | Psh29-R | GTCCGTAGTGATGTGAACCT | 60 |
|  | 2 | Psh74-F | HEX - AACAGCTACGAGTCTCAGGT | 60 |
|  |  | Psh74-R | TATGCCTGCCTAAACCTACT | 60 |
|  | 3 | Psh130-F | FAM - CAAACCTTATTTCATCAGCC | 60 |
|  |  | Psh130-R | TGTCGTTGAGACAACTCGT | 60 |
|  | 4 | Psh236-F | TAMRA - ATCTGTTCAGTAGCTTTGCC | 60 |
|  |  | Psh236-R | CAGGCCTCTAAGAAAGTTTG | 60 |
|  | 5 | Psh133-F | HEX-GCTGTCAATAGGGAGACAAG | 60 |
|  |  | Psh133-R | CCTTTAGTTTGTTTGGGTTG | 60 |
|  | 6 | Psh255-F | FAM - GCCAGTTCATAGACTCCTGA | 60 |
|  |  | Psh255-R | TCCTAACTGCAGGGTAATTG | 60 |
|  | 7 | Psh422-F | TAMRA - GCACAGGTACTCCATTCACT | 60 |
|  |  | Psh422-R | CATCAACAACTTCGAAACG | 60 |
|  | 8 | Psh463-F | HEX - GTTGGAGGTGCAGATGTTAC | 55 |
|  |  | Psh463-R | CCTCTTCCTTTCTTATGCCT | 55 |
|  | 9 | Psh493-F | FAM - GTGACTAGCACCATAAAGGC | 55 |
|  |  | Psh493-R | ATGTCTGGGAAGATTGTTTG | 55 |
|  | 10 | Psh146-F | TAMRA-TAGGTGTCTGACTTCACCCT | 55 |
|  |  | Psh146-R | TCTTCTCTTCTGAGACTGCC | 55 |
|  | 11 | Psh118-F | HEX - GGTGGTTCTCTGGAGTGTTG | 60 |
|  |  | Psh118-R | TGGTCCTCCAGTCTCACAGC | 60 |
|  | 12 | Psh131-F | FAM - TATTCCCTTTCCTACTCCT | 55 |
|  |  | Psh131-R | AACAGACCCATGATAAGAGC | 55 |
