# Supplementary material for: Long-term sky islands generate highly divergent lineages of a narrowly distributed stream salamander (Pachyhynobius shangchengensis) in mid-latitude mountains of East Asia
Source: BMC Evol Biol. 2019 Jan 3;19:1. doi: 10.1186/s12862-018-1333-8 (PMC6318985; doi:10.1186/s12862-018-1333-8)
Supplement: Supplementary file 2 — Table S2. Prior and hyperpriors used in Msvar based on microsatellite data. (DOCX 17 kb) [file 12862_2018_1333_MOESM2_ESM.docx]

**Table S2** Prior and hyperpriors used in Msvar based on microsatellite data.

|  | Prior | | | | | | | |  | Hyperpriors | | | | | | | |
| --- | --- | --- | --- | --- | --- | --- | --- | --- | --- | --- | --- | --- | --- | --- | --- | --- | --- |
| Run | *N*_0_M | *N*_0_V | *N*_1_M | *N*_1_V | tM | tV | mM | mV |  | *N*_0_M | *N*_0_V | *N*_1_M | *N*_1_V | tM | tV | mM | mV |
| 1 | 4 | 1 | 4 | 1 | 5 | 1 | -4 | 1 |  | 4 | 1 | 4 | 1 | 5 | 1 | -4 | 0.25 |
| 2 | 5 | 2 | 3 | 2 | 5 | 1 | -4 | 1 |  | 5 | 2 | 3 | 2 | 5 | 1 | -4 | 0.25 |
| 3 | 4 | 1 | 5 | 1 | 5 | 2 | -4 | 1 |  | 4 | 1 | 5 | 2 | 5 | 2 | -4 | 0.25 |
| 4 | 3 | 1 | 5 | 1 | 6 | 1 | -4 | 1 |  | 4 | 1 | 5 | 2 | 5 | 2 | -4 | 0.25 |

Note. *N*_0_, Current effective population size; *N*_1,_ ancestral effective population size; t, time in years of the change in effective population size; m, mutation rate. For each parameter, the mean (M) and variance (V) in the Msvar input are shown. The variance of the means and of the variances in the hyperpriors of all parameters were in all simulations 0 and 0.5, respectively. These four models were applied to each population analysis.
