# Supplementary figures and images for: Long-term sky islands generate highly divergent lineages of a narrowly distributed stream salamander (Pachyhynobius shangchengensis) in mid-latitude mountains of East Asia
Source: BMC Evol Biol. 2019 Jan 3;19:1. doi: 10.1186/s12862-018-1333-8 (PMC6318985; doi:10.1186/s12862-018-1333-8)

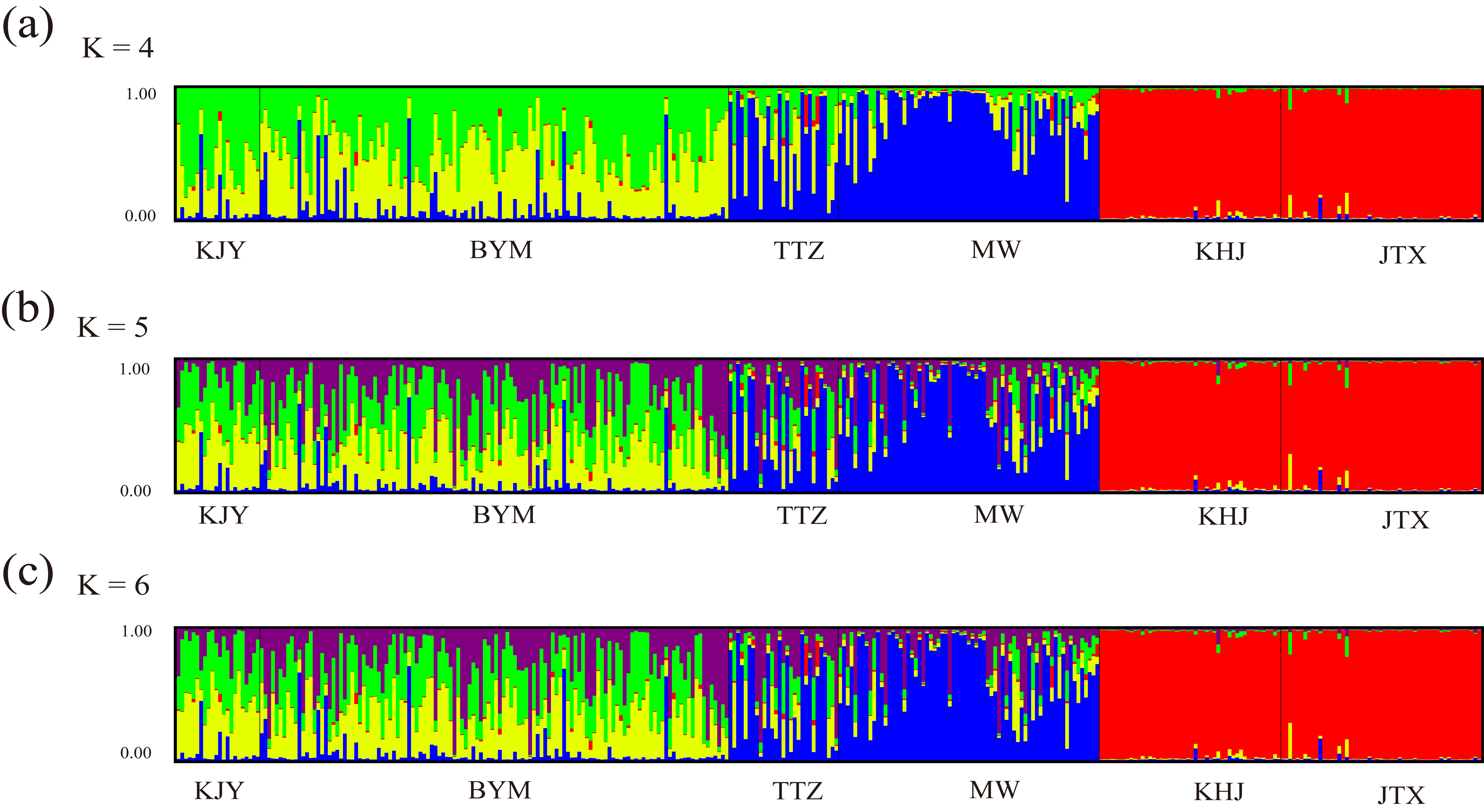

Supplement: Supplementary file 3 — Figure S1. STRUCTURE clustering results for K = 4, 5 and 6. (TIF 2521 kb) [file 12862_2018_1333_MOESM3_ESM.tif]

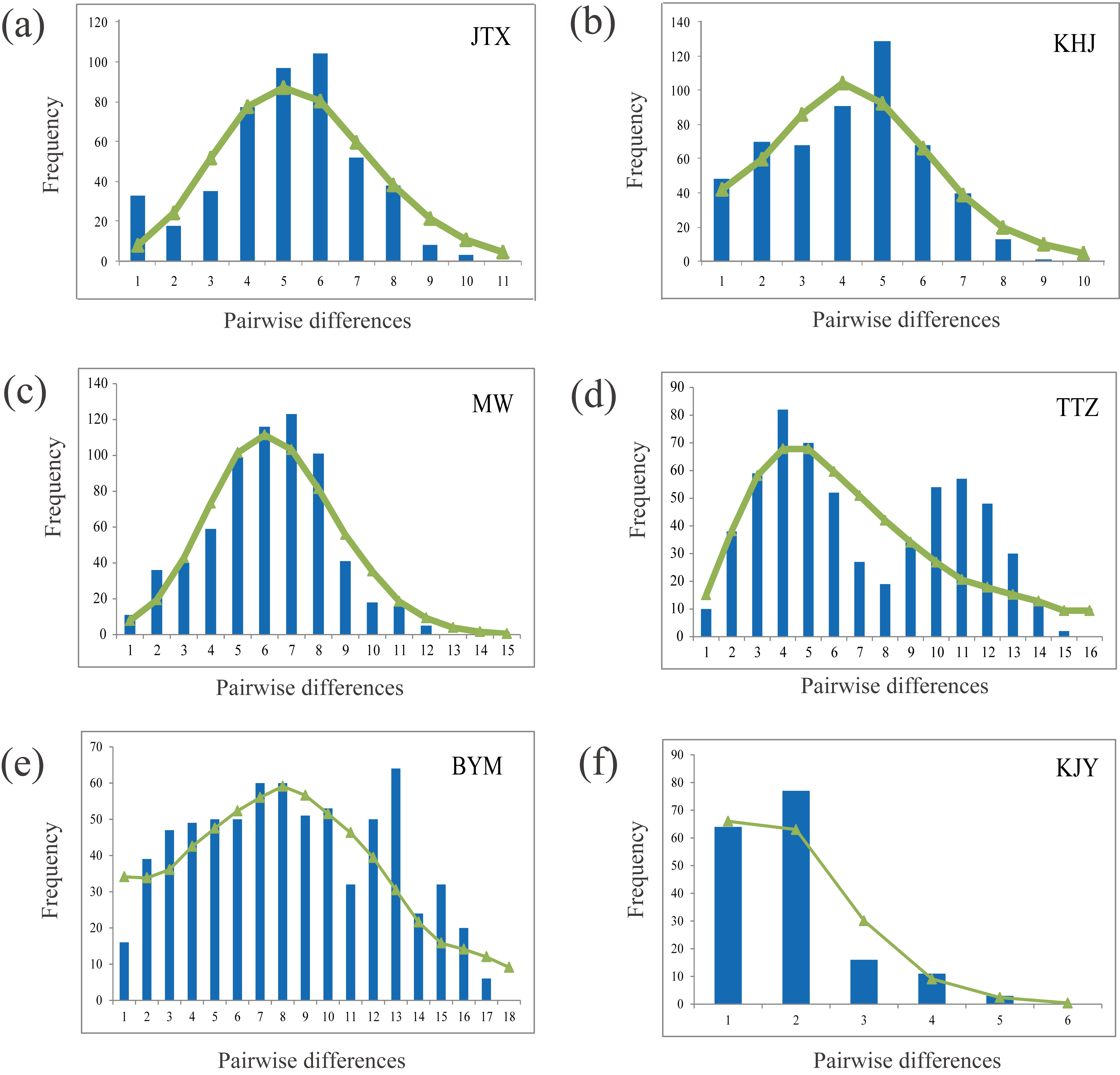

Supplement: Supplementary file 6 — Figure S2. Mismatch distributions analyses for the six populations of P. shangchengensis based on the mtDNA sequence data (a–f). The two KJY individuals clustering with BYM were excluded from the demographic history analyses. The x coordinate represents the number of differences between pairs of sequences and the y coordinate represents the frequencies of pairwise differences. The blue histograms are the observed frequencies of pairwise divergences among sequences and the green line refers to the expected shape of the distribution under the model of population expansion. (TIF 2468 kb) [file 12862_2018_1333_MOESM6_ESM.tif]

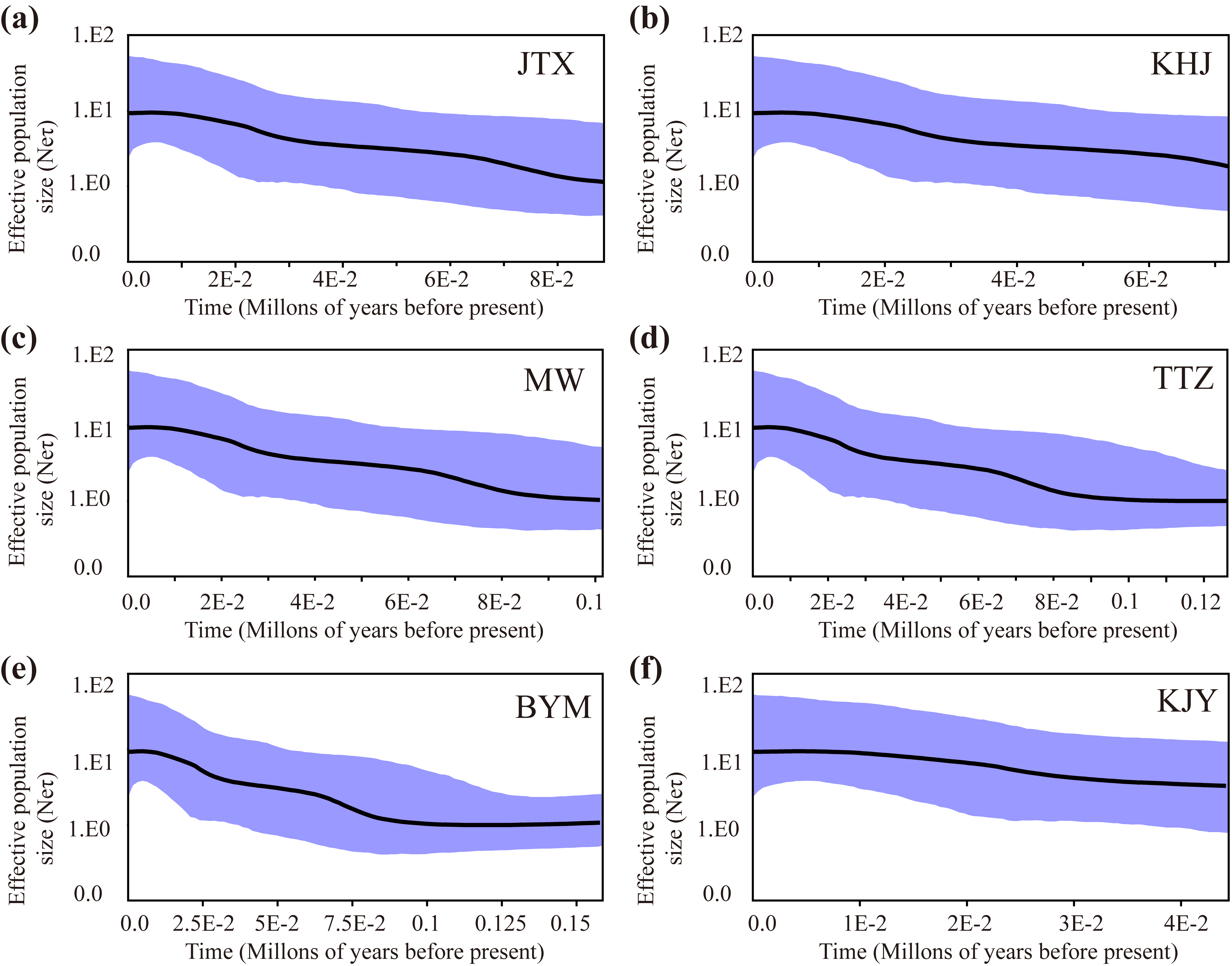

Supplement: Supplementary file 8 — Figure S3. Bayesian skyline plot of effective population size in P. shangchengensis based on the mtDNA data (a–f). The BSP analyses of the KJY population was calculated excluding the individuals with two haplotypes clustered more closely to the BYM population. The x-axis indicates time in Mya BP, and the y-axis indicates the effective population size in units of Neτ (the product of effective population size and generation time in Mya). The blue areas represent 95% highest posterior density. Time is expressed in million years. (TIF 2011 kb) [file 12862_2018_1333_MOESM8_ESM.tif]
