# Supplementary material for: Long-term sky islands generate highly divergent lineages of a narrowly distributed stream salamander (Pachyhynobius shangchengensis) in mid-latitude mountains of East Asia
Source: BMC Evol Biol. 2019 Jan 3;19:1. doi: 10.1186/s12862-018-1333-8 (PMC6318985; doi:10.1186/s12862-018-1333-8)
Supplement: Supplementary file 4 — Table S4. Multiple runs for inferring the number of populations of P. shangchengensis with Geneland. Bold indicates the highest average posterior probability. (DOCX 16 kb) [file 12862_2018_1333_MOESM4_ESM.docx]

**Table S4**. Multiple runs for inferring the number of populations of *P. shangchengensis* with Geneland. Bold indicates the highest average posterior probability.

| Run | Modal number | Average log posterior probability density | % of modal number |
| --- | --- | --- | --- |
| 1 | 6 | 4802.38 | 68 |
| 2 | 6 | 4734.60 | 88.7 |
| 3 | 6 | 4640.35 | 56.8 |
| 4 | 6 | 4678.37 | 60.8 |
| 5 | 6 | 4683.37 | 76.2 |
| 6 | 6 | 4611.23 | 89.3 |
| 7 | 6 | 4650.98 | 80.6 |
| 8 | 6 | 4734.76 | 67.8 |
| **9** | **6** | **4304.76** | **93.8** |
| 10 | 6 | 4939.58 | 63.8 |

-
