# Supplementary material for: Long-term sky islands generate highly divergent lineages of a narrowly distributed stream salamander (Pachyhynobius shangchengensis) in mid-latitude mountains of East Asia
Source: BMC Evol Biol. 2019 Jan 3;19:1. doi: 10.1186/s12862-018-1333-8 (PMC6318985; doi:10.1186/s12862-018-1333-8)
Supplement: Supplementary file 5 — Table S3. Results of hierarchical AMOVA based on mtDNA and microsatellites. Groups are set as JTX and KHJ, MW and TTZ, BYM and KJY. (DOCX 17 kb) [file 12862_2018_1333_MOESM5_ESM.docx]

**Table S3** Results of hierarchical AMOVA based on mtDNA and microsatellites. Groups are set as JTX and KHJ, MW and TTZ, BYM and KJY.

|  | d. f. | SS | VC | %V | Φ | *P* values |
| --- | --- | --- | --- | --- | --- | --- |
| **MtDNA** |  |  |  |  |  |  |
| Among groups | 2 | 4927.463 | 30.035 | 61.02 | 0.610 | 0.062 |
| Among populations within groups | 3 | 1601.844 | 16.653 | 33.83 | 0.868 | 0.000 |
| Within populations | 187 | 473.999 | 2.535 | 5.15 | 0.949 | 0.000 |
| **Microsatellite** |  |  |  |  |  |  |
| Among groups | 2 | 62.794 | 0.109 | 7.75 | 0.077 | 0.063 |
| Among populations within groups | 3 | 13.914 | 0.039 | 2.80 | 0.030 | 0.000 |
| Within populations | 684 | 861.982 | 1.260 | 89.46 | 0.105 | 0.000 |

Note. d. f., degrees of freedom; SS, sum of squares; VC, variance component; %V, percent of variance.
