# Supplementary material for: Long-term sky islands generate highly divergent lineages of a narrowly distributed stream salamander (Pachyhynobius shangchengensis) in mid-latitude mountains of East Asia
Source: BMC Evol Biol. 2019 Jan 3;19:1. doi: 10.1186/s12862-018-1333-8 (PMC6318985; doi:10.1186/s12862-018-1333-8)
Supplement: Supplementary file 7 — Table S5. Results of demographic statistics of P. shangchengensis based on mtDNA data. (DOCX 18 kb) [file 12862_2018_1333_MOESM7_ESM.docx]

**Table S5** Results of demographic statistics of *P. shangchengensis* based on mtDNA data.

|  | KJY | BYM | TTZ | MW | KHJ | JTX | Total |
| --- | --- | --- | --- | --- | --- | --- | --- |
| SSD | 0.013 | 0.005 | 0.011 | 0.004 | 0.008 | 0.009 | 0.018 |
| Model (SSD) *p*-value | 0.340 | 0.770 | 0.330 | 0.250 | 0.430 | 0.200 | 0.383 |
| Raggedness index | 0.136 | 0.008 | 0.012 | 0.017 | 0.028 | 0.030 | 0.0302 |
| Raggedness *p*-value | 0.300 | 0.960 | 0.670 | 0.340 | 0.610 | 0.310 | 0.587 |

Note: SSD, sum of square deviation (goodness-of-fit to a simulated population expansion); Raggedness: raggedness index.
