# Supplementary material for: Long-term sky islands generate highly divergent lineages of a narrowly distributed stream salamander (Pachyhynobius shangchengensis) in mid-latitude mountains of East Asia
Source: BMC Evol Biol. 2019 Jan 3;19:1. doi: 10.1186/s12862-018-1333-8 (PMC6318985; doi:10.1186/s12862-018-1333-8)
Supplement: Supplementary file 9 — Table S6. Results from Principal Components Analysis on environmental variables used in comparison of environments among occurrence localities for P. shangchengensis and the relative contribution of each variable to the niche model. (DOCX 16 kb) [file 12862_2018_1333_MOESM9_ESM.docx]

**Table S6** Results from Principal Components Analysis on environmental variables used in comparison of environments among occurrence localities for *P. shangchengensis* and the relative contribution of each variable to the niche model.

| Variable | PC1 | PC2 | Percent Contribution |
| --- | --- | --- | --- |
| Bio 3 | 0.868 | 0.337 | 0.2 |
| Bio 5 | 0.956 | 0.185 | 69.1 |
| Bio 8 | 0.953 | 0.184 | 5.5 |
| Bio 9 | 0.610 | 0.694 | 18.3 |
| Bio 13 | –0.907 | 0.169 | 4 |
| Bio 14 | –0.934 | 0.337 | 0.4 |
| Bio 15 | 0.833 | –0.499 | 0 |
| Bio 18 | –0.967 | 0.191 | 2.5 |
| Eigenvalue | 6.27 | 1.09 |  |
| %Variance explained | 78.42 | 13.64 |  |
